# PMMA-Based Continuous Hemofiltration Modulated Complement Activation and Renal Dysfunction in LPS-Induced Acute Kidney Injury

**DOI:** 10.3389/fimmu.2021.605212

**Published:** 2021-04-01

**Authors:** Alessandra Stasi, Rossana Franzin, Chiara Divella, Fabio Sallustio, Claudia Curci, Angela Picerno, Paola Pontrelli, Francesco Staffieri, Luca Lacitignola, Antonio Crovace, Vincenzo Cantaluppi, Davide Medica, Claudio Ronco, Massimo de Cal, Anna Lorenzin, Monica Zanella, Giovanni B. Pertosa, Giovanni Stallone, Loreto Gesualdo, Giuseppe Castellano

**Affiliations:** ^1^ Nephrology, Dialysis and Transplantation Unit, Department of Emergency and Organ Transplantation, University of Bari “Aldo Moro”, Bari, Italy; ^2^ Department of Interdisciplinary Medicine, University of Bari “Aldo Moro”, Bari, Italy; ^3^ Veterinary Surgery Unit, Department of Emergency and Organ Transplantation, University of Bari, Bari, Italy; ^4^ Nephrology and Kidney Transplantation Unit, Department of Translational Medicine, University of Piemonte Orientale (UPO), Novara, Italy; ^5^ Department of Nephrology, Dialysis and Transplantation, San Bortolo Hospital, Vicenza, Italy; ^6^ International Renal Research Institute of Vicenza (IRRIV), Vicenza, Italy; ^7^ Department of Medicine - DIMED, University of Padova, Padova, Italy; ^8^ Nephrology, Dialysis and Transplantation Unit, Department of Medical and Surgical Science, University of Foggia, Foggia, Italy

**Keywords:** LPS-induced AKI, PMMA-CVVH treatment, immunological dysfunction, complement modulation, gene expression profile

## Abstract

Sepsis-induced acute kidney injury (AKI) is a frequent complication in critically ill patients, refractory to conventional treatments. Aberrant activation of innate immune system may affect organ damage with poor prognosis for septic patients. Here, we investigated the efficacy of polymethyl methacrylate membrane (PMMA)-based continuous hemofiltration (CVVH) in modulating systemic and tissue immune activation in a swine model of LPS-induced AKI. After 3 h from LPS infusion, animals underwent to PMMA-CVVH or polysulfone (PS)-CVVH. Renal deposition of terminal complement mediator C5b-9 and of Pentraxin-3 (PTX3) deposits were evaluated on biopsies whereas systemic Complement activation was assessed by ELISA assay. Gene expression profile was performed from isolated peripheral blood mononuclear cells (PBMC) by microarrays and the results validated by Real-time PCR. Endotoxemic pigs presented oliguric AKI with increased tubulo-interstitial infiltrate, extensive collagen deposition, and glomerular thrombi; local PTX-3 and C5b-9 renal deposits and increased serum activation of classical and alternative Complement pathways were found in endotoxemic animals. PMMA-CVVH treatment significantly reduced tissue and systemic Complement activation limiting renal damage and fibrosis. By microarray analysis, we identified 711 and 913 differentially expressed genes with a fold change >2 and a false discovery rate <0.05 in endotoxemic pigs and PMMA-CVVH treated-animals, respectively. The most modulated genes were Granzyme B, Complement Factor B, Complement Component 4 Binding Protein Alpha, IL-12, and SERPINB-1 that were closely related to sepsis-induced immunological process. Our data suggest that PMMA-based CVVH can efficiently modulate immunological dysfunction in LPS-induced AKI.

## Introduction

Sepsis represents a very relevant problem in critical ill patients without specific and efficient therapies (1, 2). Clinical and experimental studies describe sepsis as a systemic disease characterized by an early overwhelmed inflammatory response to a primary insult leading to multiple organ failure and death (2). This systemic response is accompanied by excessive activation of the Complement and coagulation system with cytokine storm and endothelial dysfunction triggered both by the pathogen itself and by damaged tissue (2, 3).

One of the severely affected organs is the kidney and sepsis-associated acute kidney injury (AKI) has become a widespread and important problem in the ICU (1, 4). The pathophysiology of AKI during sepsis is complex and is associated with permanent alterations of renal resident cells that may promote chronic renal failure (5–7). A broad range of mediators have been identified as responsible for sepsis-related tissue injury, with cellular and soluble components of innate immunity. Among them, Complement system play a critical role since the uncontrolled Complement activation can affect organ damage with poor prognosis for septic patients (2, 3).

Recently, it has been shown that the pathogenetic mechanisms of COVID-19 disease reflects the maladaptive host immune response observed in sepsis (8). Indeed, SARS-CoV-2 infection and the damaged tissues lead to an uncontrolled systemic inflammatory response with increased complement activation (9), abnormal coagulation, endothelial dysfunction, and subsequent multi-organ damage (10). Like sepsis, the occurrence of AKI was considered the most commonly reported co-morbidity of COVID-19 disease (11–13). Therefore it is reasonable that advances in septic field could have a great impact in the pathophysiology and mechanisms of COVID-19 disease (14).

Gram-negative infections are a common variant of sepsis that occurs in 15% of diagnosed patients frequently inducing circulatory collapse and death within hours (2). This is mainly due to the release of their outer wall component, named lipopolysaccharide (LPS) or endotoxin that can activate a wide variety of cells through interaction with specific pattern recognition receptors (PRRs), such as toll-like receptors (TLRs) (15). In the kidney, LPS binds TLR4 and activates tubular, endothelial cells (16) and pericytes (17). Endotoxin requires a carrier protein, known as LPS-binding protein (LBP) that brings LPS to TLR4 and maximizes the activation of the downstream intracellular signaling (5, 6, 18).

During the last three decades numerous clinical trials based on the employment of anti-inflammatory mediators, specific inhibitors of LPS–TLR4 signaling (19) have failed to improve outcome in septic subjects (20). Although these approaches may in part modify the systemic inflammatory response, the protracted immunosuppression may lead to immunoparesis with the acquisition of a secondary infection in patients (20, 21). Interestingly, therapeutic strategies based on blood purification have been developed for the treatment of sepsis-induced AKI *via* an extracorporeal circuit through a device (sorbent), modulating the disequilibrium between pro and anti-inflammatory mediators (22). The use of polymethyl methacrylate (PMMA) membrane hemofilter could improve septic patients outcome, due to excellent biocompatibility and adsorptive properties of middle- to high molecular weight substances (23, 24). Here we investigate the capacity of PMMA-based-Continuous Veno-Venous Hemofiltration (PMMA-CVVH) in modulating innate immune activation in a swine model of LPS-induced AKI.

## Materials and Methods

### Animal Model

Animal study was performed in domestic swine, after approval by the ethical committee of the Italian Ministry of Education, University and Research (MIUR) (Prot. n 823/2016-PR). Briefly, LPS-induced AKI was induced by intravenous infusion of a saline solution containing 300 μg/kg of LPS (lipopolysaccharide membrane of *Escherichia coli*), as previously described (25).

The animals were randomized into four groups: healthy (healthy pigs, n = 7), LPS (endotoxemic pigs, n = 7), PS-CVVH (Polysulfone CVVH-treated endotoxemic pigs, n = 7; TORAYLIGHT NS-S 1.8, Toray Medical Co, Japan), and PMMA-CVVH (Polymethylmethacrylate CVVH-treated endotoxemic pigs, n = 7; HEMOFEEL CH 1.8W-Toray Medical Co, Japan).

HEMOFEEL CH-1.8W is a non-ionic PMMA membrane with an effective surface area of 1.8 square meters, an internal hollow fiber diameter of 240 μm and a wall thickness of 30 μm. This new membrane recently obtained the CE mark and is available for the clinical use in Europe. An ultrafiltration rate (UFR) of 30 ml/h/mmHg was measured in standard experimental setting condition with a Qb = 200 ml/min. The treatments in PMMA-CVVH group (n = 7) or PS-CVVH group (n = 7) were performed with a CVVH heparin-based treatment in pre-dilution modality using a dedicated machine EQUAsmart^®^ (Medica S.p.A, Medolla, Italy). The Blood Flow rate was set at 150 ml/min with an ultrafiltration rate set at 2,000 ml/h using PrismaSol^®^ solution (Baxter Renal Products, Inc, USA) as replacement fluid. The anticipated net weight loss was set at zero and neither plasma water nor albumin compensation were necessary at the end of 7 h of treatments.

In both PS and PMMA group, the CVVH treatments started after 3 h from LPS infusion with a duration of 7 h. Hemodynamic parameters included mean arterial pressure (mmHg) were automatically registered and recorded every 30 s (25).

Animals were sacrificed after 24 h from LPS/saline infusion with an overdose of IV propofol, immediately followed by a 10-ml IV bolus of an oversaturated solution of potassium chloride (2 mEq/ml, Galenica Senese, srl, Italy).

In total, twenty-eight animals were examined in this study. Three animals (two animals of the LPS group and one animal of the PS group) died because of severe low blood pressure before completing the study protocol. The data of these animals were included until the last useful measurement (LPS animals: 6 and 8 h after LPS injection; PS animal: 12 h after LPS injection). Therefore, Healthy group and PMMA group, n = 7 for all time points; LPS group, T0-T3 n = 7 and T10-T24 n = 5; PS group, T0-T3-T10 n = 7 and T24 n = 6.

### Collection of Samples

At sacrifice, kidneys were collected from all animals and processed by using standard procedures as previously described (6). Urine samples were collected from all animals and urinary output was measured and recorded every hour. Swine sera were collected at baseline, at intermediate time points and at 24 h from an arterial blood catheter. Renal function was assessed by serum creatinine and monitoring urinary output.

### Histologic Analysis

Renal tissues were processed for histologic staining [hematoxylin and eosin (HE) and Masson’s trichrome, (Millipore Sigma)]. Then, digital slides were analyzed and acquired by the AperioScanScope CS2 device (Aperio, Vista, CA, USA) with 20× or 40× magnification as previously described (25). HE and Masson trichrome staining were performed to evaluate histological injury and fibrosis. Tubular and glomerular injury was scored semi quantitatively by two blinded observers who examined at least 20 visual field of each kidney section using criteria of previously published studies (26–30). The tubular damage was defined by evident signs of tubular dilation, tubular atrophy, tubular epithelial cell necrosis, and cast formation. The score of tubular lesions was assessed by the following score: 0 = normal kidney without tubular injury; 1 = <10% of tubules injured; Score 2: 10–25% of tubules injured; Score 3: 25–50% of tubules injured; Score 4: 50–74% of tubules injured; Score 5: >75% of tubules injured (26). The glomerular lesion score: 0 = normal glomeruli; 1 = mild deposition of fibrin and reduced number of capillaries in a few glomeruli; 2 = moderate fibrin deposition and reduced number of capillaries in numerous glomeruli; 3 = marked fibrin deposition and reduced number of capillaries in numerous glomeruli; 4 = marked fibrin deposition and reduced number of capillaries in numerous glomeruli and glomerulosclerosis; 5 = glomerulosclerosis (26). The score index in each animal was expressed as a mean value of all scores obtained. Both tubular and glomerular pathological score of each group was expressed as median ± IQR (Healthy and PMMA T24 group, n = 7, LPS T24 n = 5, PS T24 n = 6). Tubulointerstitial fibrosis was assessed by Masson’s trichrome staining and the green-stained area was calculated using Adobe Photoshop software (Adobe, San Jose, CA, USA) and expressed it as positive pixel/total pixel (25).

### Confocal Laser Scanning Microscopy

Swine paraffin-embedded renal sections were stained for PTX3 (clone MNB4, rat, Exira Life Sciences In., Larsen, Switzerland) and C5b-9 (mouse, Abcam, Cambridge, UK). Tissue sections were deparaffinized through xylene and alcohol and underwent epitope retrieval through three microwave (750 W) cycles of 5 min in citrate buffer (pH = 6). Then, they were incubated with specific blocking solution, primary antibodies (anti-PTX3 1:100 and anti-C5b-9 1:50) and the corresponding secondary antibodies (Alexa Flour 488, Molecular Probes, Eugene, OR, USA). All sections were counterstained with TO-PRO-3 (Molecular Probes) or DAPI (Thermo Fisher, MA, USA) and mounted with Fluor mount. Negative controls were prepared omitting the primary antibody. Image acquisition was performed by confocal microscope Leica TCS SP8 (Leica, Wetzlar, Germany). Both PTX3 and C5b-9 fluorescence signals were quantified by confocal microscope Leica TCS SP2 software and expressed as area fraction (percentage).

### LBP, sCD40, and sCD40L Serum ELISA

Serum LBP levels were measured by ELISA kit from HycultBiotech (Uden, Netherlands) as well as sCD40 (LifeSpan Biosciences Inc, Seattle, WA, USA) and CD40 ligand (MyBioSource, San Diego, USA).

### Assessment of the Activity of Classical, Lectin, and Alternative Complement Pathways

Swine sera were analyzed using the Wieslab kit (WIESLAB Complement System Screen COMPL 300, Euro-Diagnostica) as previously described (31).

### PBMC Isolation and RNA Extraction

Thirty ml of whole blood was harvested at baseline (T0, before LPS infusion) and at the end of the study (T24). PBMCs were isolated by density separation over a Ficoll-Paque (GE Healthcare, Uppsala, Sweden). Total RNA was extracted automatically and qualitatively and quantitatively analyzed through Agilent 2100 Bioanalyzer (Agilent Technologies, Santa Clara, CA, USA). Only samples with good quality characterized by RIN >8 were used in the microarray experiment.

### Microarray Experiment

For transcriptomic profiling, labeled cRNA was obtained using the Low Input Quick Amp Labeling Kit (Agilent Technologies) from RNA samples of pigs at baseline (respectively five T0 LPS group and three PMMA T0 group), four endotoxemic animals (T24 LPS group), and four PMMA-CVVH treated-endotoxemic pigs (T24 PMMA group). However, one sample of LPS T24 group was excluded due to technical problem. Gene expression data were obtained using Agilent Feature Extraction software (v.10.7.3). The Agilent microarray data are Minimum Information About a Microarray Experiment (MIAME) compliant, and raw data have been deposited in the database of the European Bioinformatics Institute (EMBL-EBI) and are accessible through Experiment ArrayExpress accession number E-MTAB-9145. Gene expression analysis was performed by Genespring GX 7.2 (Agilent, Santa Clara, CA, USA), using a false discovery rate (FDR) <0.05 and a fold change (FC) >2.

### Real-Time PCR Analysis

Real-time PCR was performed to validate microarray gene expression data of five differentially modulated genes [Granzyme B (GZMB), Complement factor B (CFB), Complement Component 4 Binding Protein Alpha (C4BPA), SERPIN B1, and IL-12]. These experiments were performed on the same samples used in the microarray experiments and extended to the other samples of each group not used in microarray analysis. Total RNA (500 ng) was used in a reverse transcription reaction by using the iScript cDNA Synthesis Kit (Bio-Rad, Hercules, CA, USA) according to the manufacturer’s instructions. Real-time quantitative PCR was performed on the Light Cycler@96 (Roche) by using GZMB, CFB, C4BPA, SERPINB1, and IL-12 primers (validated primers for swine genome: GZMB, Assay ID: qSscCED0016345; CFB, Assay ID: qSscCED0017919; C4BPA, Assay ID: qSscCID0003068; SERPINB1, Assay ID: qSscCID0012570; IL-12B, Assay ID: qSscCED0008922; Bio-Rad) in combination with SsoAdvanced™ Universal SYBR^®^ Green Supermix (Biorad). The relative amounts of mRNA were normalized to β-actin mRNA as the housekeeping gene. Data were analyzed using the ΔΔC_t_ method.

### Statistical Analysis

Identification of genes differentially expressed between LPS and PMMA treatments was carried out with FDR method of Benjamini-Hochberg (32) and gene probe sets were filtered on the basis of the false discovery rate, FDR (adjusted-P value with multiple testing on 1,000 permutations), and fold-change (28). Fold change filter was set to 2-fold in each comparison. Only genes that were significantly modulated (adjusted-P value <0.05 and fold-change >2) were considered for further analysis. For real-time PCR, data were presented as mean ± standard deviation (SD) (Healthy and PMMA group, T0 and T24 n = 7; LPS group, T0 n = 7 and T24 n = 5; PS group, T0 n = 7 and T24 n = 6) and compared by paired or unpaired Student t-test as appropriate.

In the other analyses, the results were expressed as median ± interquartile range (IQR) (Healthy and PMMA group, n = 7 for all time points; LPS group, T0-T3 n = 7 and T10-T24 n = 5; PS group, T0-T3-T10 n = 7 and T24 n = 6) and compared with a Mann–Whitney U test. All data (results) were analyzed using GraphPad Prism 5.0 (GraphPad software, Inc., San Diego, CA, USA). A *p-value <*0.05 was considered statistically significant.

## Results

### Improvement of LPS-Induced AKI by PMMA-Based CVVH Treatment

**Figure 1:**
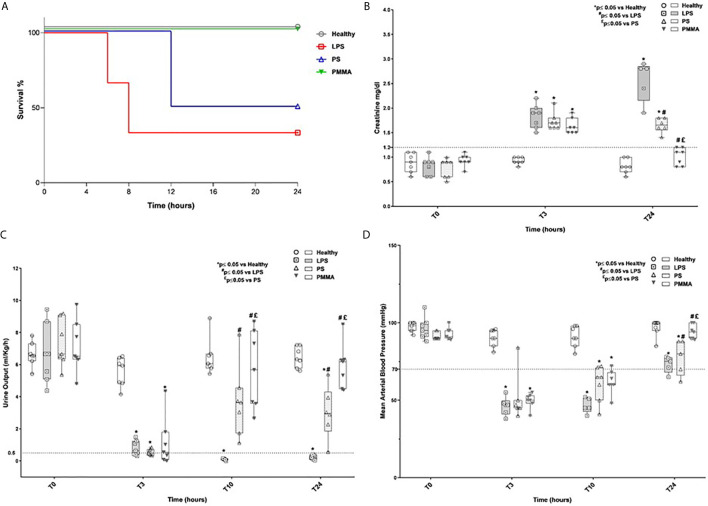
Survival rate, renal function, oligo-anuria, and hypotension in a swine model of LPS-induced AKI. **(A)** Three animals (two animals of the LPS group and one animal of the PS group) died because of severe low blood pressure before completing the study protocol. **(B, C)** Endotoxemic pigs developed AKI with time-dependently increased creatinine serum levels and reduced urinary output. PMMA-CVVH treatment significantly reversed LPS effects (^#^p < 0.05 *vs* LPS, and ^£^p < 0.05 *vs* PS). **(D)** After LPS infusion, animals presented hypotension and PMMA-CVVH treatment recovered MAP levels more than PS-CVVH treatment. **(B–D)** PS-CVVH treatment was no effective to recover creatine level, urine output, and arterial blood pressure to baseline median of healthy group. Data were obtained as described in the *Methods* section and expressed as median ± IQR of at least five pigs for each group (Healthy group and PMMA group, n = 7 for all time points; LPS group, T0-T3 n = 7, T10-T24 n = 5; PS group, T0-T3-T10 n = 7 and T24 n = 6). Statistically significant differences were assessed by the Mann–Whitney test (*p < 0.05 *vs* Healthy, ^#^p < 0.05 *vs* LPS, and ^£^p < 0.05 *vs* PS).

We first analyzed animal survival in 24 h by Kaplan-Meier curve (**Figure 1A**). No significant statistical difference was observed by log-rank, in the four groups. Three animals (two animals of the LPS group and one animal of the PS group) died because of severe low blood pressure before completing the study protocol. Next, we found that AKI was successfully induced in LPS and treated animals (**Figures 1B–D**). As previously showed (25), LPS injection led to a time-dependent increase of serum creatinine, with significant reduction in urinary output (ml/kg/h) (**Figures 1B, C**) and hypotension (**Figure 1D**) compared to healthy group. The presence of oliguria (<0.5 ml/kg/h), rise in serum creatinine (>1.2 mg/dl), and hypotension (<70 mm/Hg) well characterized renal damage in endotoxemic animals.

Interestingly, PMMA-CVVH significantly improved 24-h creatinine level, 24-h urine output, and arterial blood pressure (**Figures 1B–D**) compared to both LPS and PS-treated group. On the contrary, we observed very limited effects by PS-CVVH treatment. Indeed, PS-CVVH was no effective to recover creatine level and urine output.

**Figure 2:**
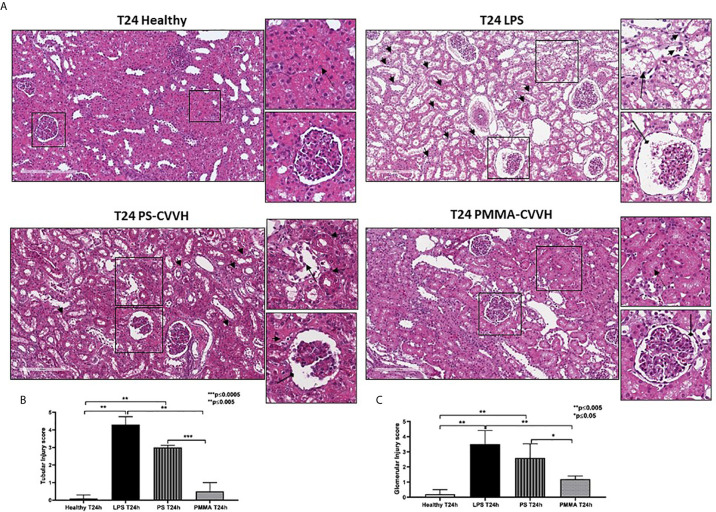
Recovery of parenchymal damage in endotoxemic animals. **(A)** H&E staining showed significant desquamation of proximal tubular epithelial cells (zoomed image, black arrow), marked fibrin deposition and loss of capillaries in numerous glomeruli, Bowman’s capsule expansion (zoomed image, black arrow with rounded tip), and interstitial inflammatory infiltrate (T24 LPS and zoomed image, small black arrows) after 24 h from LPS infusion compared to control (T24 Healthy). PS-CVVH treatment reduced inflammatory infiltrate (small black arrows) but not preserved glomerular (loss of capillaries and Bowman’s capsule expansion; zoomed image, black arrow with rounded tip) and tubular compartment from LPS injury (zoomed image, black arrow). In PS-CVVH group, few tubules did not show signs of damage and vacuolization (zoomed image, dotted arrows). Renal biopsies of endotoxemic animals after PMMA-CVVH treatment showed recovery of tubular damage (zoomed image, dotted arrow) and reduced pathological changes at glomerular level (zoomed image, black arrow with rounded tip) and inflammatory infiltrate (zoomed image, black arrows). **(B, C)** Tubular and glomerular pathological score was obtained as described in the *Methods* section and expressed as median ± IQR of at least five pigs for each group (Healthy and PMMA T24 group, n = 7, LPS T24 n = 5, PS T24 n = 6). Statistically significant differences were assessed by the Mann–Whitney test (*p < 0.05; **p < 0.005; ***p < 0.0005).

Following 24 h from LPS infusion, we observed significant morphological changes in renal parenchymal, including tubular vacuolization, epithelial flattening, necrosis, infiltration of inflammatory cells, and marked fibrin deposition with reduced number of capillaries in numerous glomeruli (**Figure 2A**, T24 LPS).

The pathological scores of renal tubular and glomerular injury in PS-CVVH group was significantly higher than those observed in healthy group (**Figures 2B, C**). The treatment by PS-CVVH was less capable to limit renal tissue damage (**Figure 2A**, T24 PS-CVVH).

Comparing with both septic and PS-CVVH pigs (**Figures 2A–C**, T24 LPS and T24 PS-CVVH), PMMA-CVVH significantly reduced tubular and glomerular damage and hampered the recruitment of inflammatory infiltrate (**Figure 2**, T24 PMMA-CVVH).

### PMMA-CVVH Treatment Reduced Collagen Deposition and Renal Fibrosis in Endotoxemic Animals

**Figure 3:**
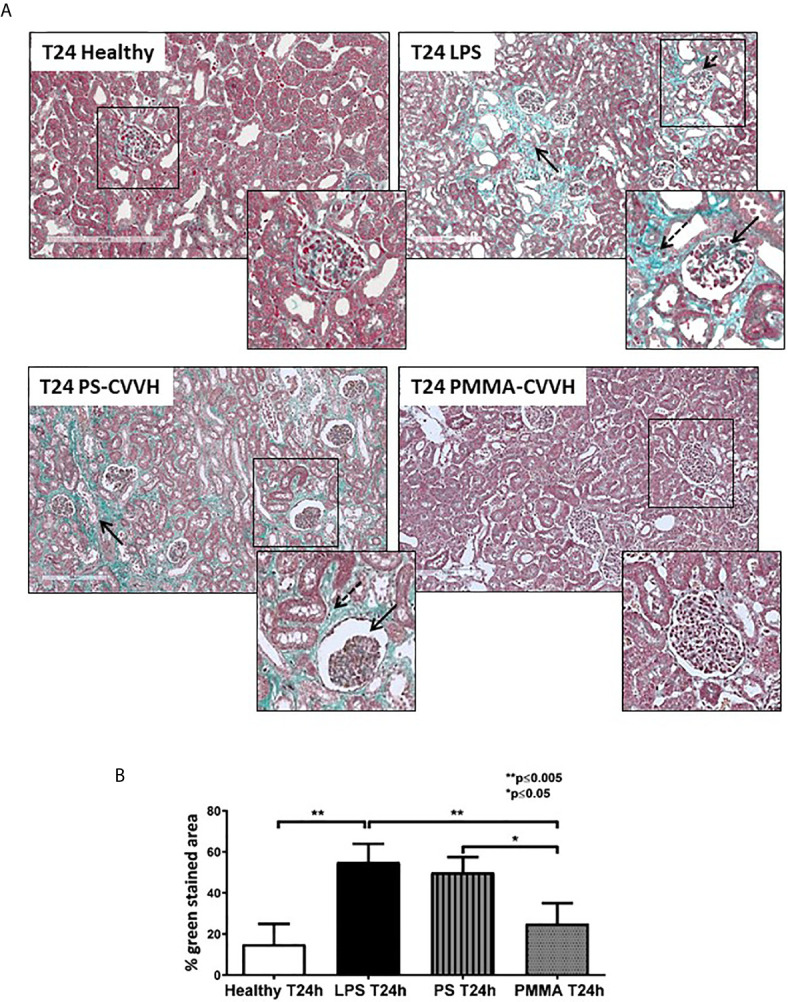
PMMA-CVVH treatment prevented early fibrosis in endotoxemic pigs. Masson trichrome staining **(A)** revealed extensive collagen deposition at the interstitial level, along capillaries (zoomed image, black arrow), and diffuse glomerular thrombi (zoomed image, dotted black arrow) in endotoxemic pigs compared with T24 of the control group (T24 Healthy). Renal biopsies after PS-CVVH treatment did not reduce collagen deposits (black arrows). Otherwise, PMMA-CVVH treatment strongly decreased collagen deposits (zoomed image, black arrows) and renal damage. **(B)**The quantitative analysis was obtained as described in the Methods section and expressed as median ± IQR of at least five pigs for each group (Healthy and PMMA T24 group, n = 7, LPS T24 n = 5, PS T24 n = 6). Statistically significant differences were assessed by the Mann–Whitney test (*p < 0.05; **p < 0.005).

The presence of glomerular thrombi and collagen deposits are considered hallmark of sepsis-induced AKI (5, 6). Compared to healthy pigs, collagen deposits significantly increased after 24 h from LPS injection thus indicating the acute development of fibrosis (**Figure 3A**, T24 LPS); PS-CVVH was not effective in counteracting the development of renal fibrosis and glomerular thrombi and injury (**Figure 2A**, T24 PS-CVVH). On the contrary, PMMA-CVVH treatment preserved renal parenchyma significantly decreasing glomerular damage and collagen deposits respect to both LPS and PS group (**Figures 3A, B**).

### Effects of PMMA-CVVH Treatment on LPS-Induced PTX-3 Deposits and Complement Activation

**Figure 4:**
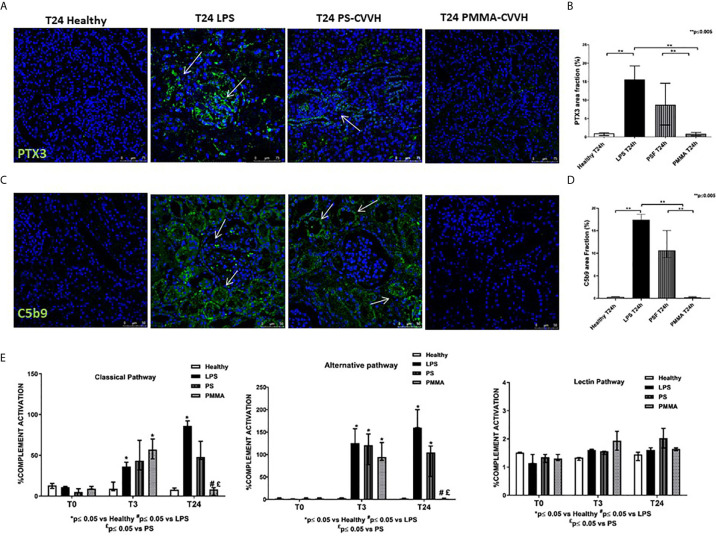
PMMA-CVVH treatment reduced PTX3 deposits and local and systemic Complement activation. **(A)** In healthy pigs, no PTX3 deposits (green) were found. A significant increase in PTX3 deposits were observed at peritubular and glomerular level (white arrows) in LPS group. PS-CVVH reduced PTX3 deposits. PMMA-CVVH treatment significantly reversed LPS effects, respect to both LPS and PS-CVVH group. Magnification 630×. The fluorescent dye To-pro 3 was used to counterstain nuclei (blue). **(B)** C5b9 deposits (green) were observed at tubular and glomerular level in endotoxemic pigs (white arrows). Renal C5b-9 staining was still detected in PS-CVVH group (white arrows). A significant decrease of C5b-9 deposits was observed after PMMA-CVVH treatment. Magnification 630×. The fluorescent dye TOPRO was used to counterstain nuclei (blue) The quantitative analyses of PTX3 **(C)** and C5b-9 staining **(D)** were obtained as described in the Methods section and expressed as median ± IQR of at least five pigs for each group (Healthy and PMMA T24 group, n = 7, LPS T24 n = 5, PS T24 n = 6). Statistically significant differences were assessed by the Mann–Whitney test (**p < 0.005). **(E)** Wieslab assay revealed systemic activation of complement classical and alternative pathway in endotoxemic swine sera after 3 and 24 h from LPS injection. PMMA-CVVH treatment significantly decreased both CP and AP (^#^p < 0.05 *vs* LPS, and ^£^p < 0.05 *vs* PS). PS-CVVH treatment did not reduce systemic complement activation. Results represent the median ± IQR of at least five pigs for each group (Healthy group and PMMA group, n = 7 for all time points; LPS group, T0-T3 n = 7, T10 -T24 n = 5; PS group, T0-T3-T10 n = 7 and T24 n = 6). Statistically significant differences were assessed by the Mann–Whitney test (*p < 0.05 *vs* Healthy, ^#^p < 0.05 *vs* LPS, and ^£^p < 0.05 *vs* PS).

PTX3 has been implicated in septic shock (33) and it can activate the classical pathway of Complement (34, 35) through C1q binding, amplifying the severity of the disease. We then analyzed PTX3 localization and distribution in our model of sepsis-induced AKI. We observed very limited PTX3 deposits in healthy pigs (**Figures 4A, B**); after 24 h from LPS infusion, PTX-3 deposits significantly increased in peritubular and glomerular capillaries. Interestingly, we found a partial reduction in PTX-3 deposits by PS-CVVH, that were virtually absent in PMMA-CVVH treated animals (**Figures 4A, B**).

Next to PTX3, we investigated the tissue activation of Complement in order to make comparison with human data (3). We evaluated the deposition of the terminal Complement complex C5b-9, using an antibody directed against the C9-neo-epitope (**Figures 4C, D**). In control animals, C5b-9 deposits were rarely present, while, in endotoxemic pigs, we found significant C5b-9 activation, mainly localized at tubule-interstitial level. Renal C5b-9 staining was still detectable in PS-CVVH group but completely absent in PMMA-CVVH treatment (**Figures 4C, D**).

We then examined systemic Complement activation by analyzing serum from septic and treated pigs (**Figure 4E**); we found that serum from LPS pigs induced a significant increase in the activation of classical and alternative pathways, with no effects on the lectin pathway, after 3 and 24 h from LPS infusion. Interestingly, PMMA-CVVH treatment was able to significantly inhibit both the classical and alternative pathways at 24 h compared to LPS and PS group. Regard to PS-CVVH, we did not detect significant differences in Complement activation compared to LPS group.

### Immunomodulatory Effects of PMMA-CVVH on Circulating Pro-inflammatory Factors

**Figure 5:**
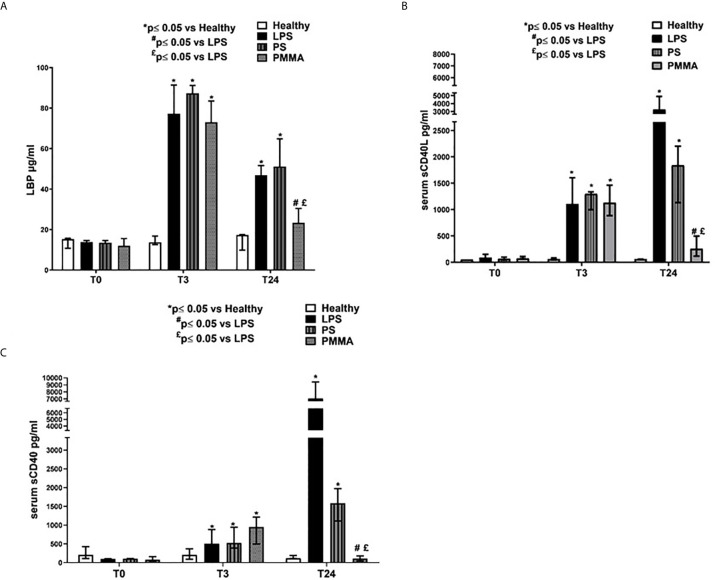
PMMA-CVVH treatment modulated TLR-4 signaling and reduced sCD40L and sCD40 sera levels. **(A)** ELISA assay showed significantly increased level of serum LBP in endotoxemic pigs after 3 and 24 h from LPS infusion respect to healthy group. A strong reduction in serum LBP levels was found after PMMA-CVVH treatment (^#^p < 0.05 *vs* LPS, and ^£^p < 0.05 *vs* PS). PS-CVVH did not modulate LBP sera levels. **(B, C)** ELISA assay showed significantly increased level of serum sCD40L **(B)** and sCD40 **(C)** in endotoxemic pigs after 3 and 24 h from LPS infusion respect to healthy group. A significant strong decrease in serum sCD40L and sCD40 levels was found after PMMA-CVVH treatment (^#^p < 0.05 *vs* LPS, and ^£^p < 0.05 *vs* PS). PS-CVVH reduced sCD40L and sCD40 sera levels but it did not reach the basal condition as with PMMA treatment. **(A–C)** The histograms represent the median ± IQR of at least five animals for each group (Healthy group and PMMA group, n = 7 for all time points; LPS group, T0-T3 n = 7, T10 -T24 n = 5; PS group, T0-T3-T10 n = 7 and T24 n = 6). Statistically significant differences were assessed by the Mann–Whitney test (*p < 0.05 *vs* Healthy, ^#^p < 0.05 *vs* LPS, and ^£^p < 0.05 *vs* PS).

We then measured LBP serum levels in endotoxemic pigs, and we observed a significant increase after 3 and 24 h from LPS infusion. Interestingly, PMMA-CVVH treatment significantly reduced LBP levels in the circulation thereby counteracting LPS signaling (**Figure 5A**). On the contrary, we found no significant difference between the PS-CVVH group and the LPS group.

In addition, we also evaluated the sCD40/CD40L axis that has been associated with development of AKI (36) (**Figures 5B, C**). sCD40L has been considered as a marker of inflammation (37) and thrombosis (38), and it is significantly increased in patients with severe sepsis (39). We found high levels of sCD40L in endotoxemic pigs after 3 and 24 h from LPS infusion. PMMA-CVVH treatment reversed LPS effects, significantly reducing sCD40L serum level compared to both endotoxemic and PS-treated animals; on the contrary, PS-CVVH treatment did not exert significant effects in sCD40L removal (**Figure 5B**). In accordance, PMMA-CVVH treatment induced a significant downregulation of sCD40 serum level (**Figure 5C**), demonstrating a protective effect of PMMA membrane in sustaining immune competence. On the contrary PS-CVVH treatment was not able to reduce sCD40 level at baseline.

### PMMA-CVVH Modulated Expression of Pro-inflammatory Genes in Swine PBMC

**Figure 6:**
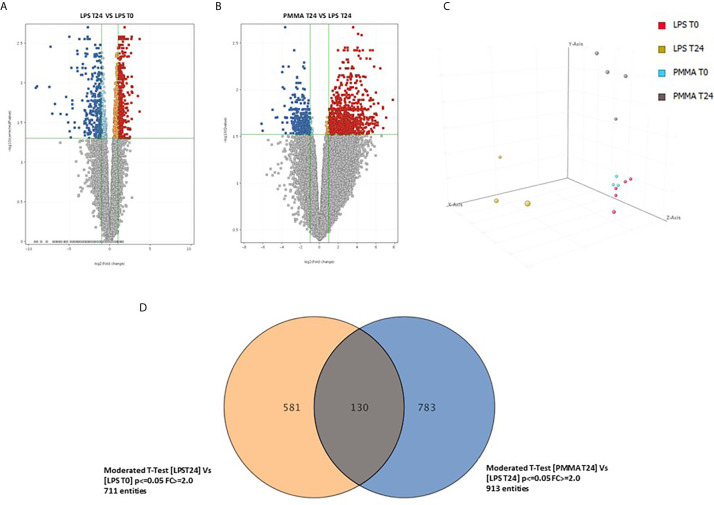
Comparison of the whole-genome gene expression profiles of swine PBMC. **(A)** Volcano plot show 711 genes significantly modulated with a FDR < 0.05 and a FC > 2; 310 genes were upregulated and 401 genes were downregulated (LPS 24 *VS* LPS T0). **(B)** Volcano plot show 913 genes significantly modulated with an FDR < 0.05 and a FC > 2; 640 genes were upregulated and 273 genes were downregulated (PMMA T24 *VS* LPS T24). **(C)** Principal component analysis 3-D diagram showed different spatial distribution of the LPS T24h and PMMA T24h pigs from the healthy cohort (LPS T0 and PMMA T0). **(D)** Venn diagram showing shared and specific genes for LPS T24 and PMMA group in swine PBMCs.

In order to identify genes specifically modulated after 24 h from LPS infusion, we compared the whole-genome gene expression profiles of swine PBMC after 24 h from LPS infusion (T24) and before LPS infusion (T0). We identified 711 genes significantly modulated with an FDR <0.05 and a FC>2 (**Figure 6A**, **Supplementary Table 1**); 310 genes were upregulated and 401 genes were downregulated. Then, we compared the PBMC gene expression profiles of PMMA-treated endotoxemic pigs (PMMA T24 group) with that of endotoxemic animals at T24 (LPS T24 group). We identified 913 genes significantly modulated with an FDR <0.05 and a FC >2 (**Figure 6B**, **Supplementary Table 2**); 640 genes were upregulated and 273 genes were downregulated. We found that the Principal Component Analysis (PCA, **Figure 6C**) could well discriminated LPS and PMMA-CVVH treated pigs from baseline gene expression (LPS T0 and PMMA T0).

Finally, we crossed the two analyses to identify genes specifically modulated by the PMMA-CVVH treatment. We found that 581 genes were modulated exclusively by LPS; interestingly, the treatment by PMMA-CVVH could modulate 783 genes after LPS infusion. However, 130 genes were modulated by LPS even in presence of PMMA treatment (**Figure 6D** and **Supplementary Tables 3–5**). Among these modulated genes, we focused on those involved in sepsis-induced immunological activation. We found two genes, GZMB and CFB, that were significantly upregulated in LPS T24 group compared to basal condition (LPS T0). GZMB belong to the family of serine proteases and is constitutively expressed in several immune cells, including cytotoxic T lymphocytes, natural killer cells, NKT cells, and γδ T cells and its augmented expression is correlated with disease severity. CFB is a necessary component of the Complement alternative pathway and an important downstream effector of TLR signaling in sepsis.

Interestingly, we observed that PMMA-CVVH treatment induced a down-regulation of GZMB and the upregulation of Complement Component 4 Binding Protein Alpha (C4BPA), SERPIN B1, and IL-12 (**Supplementary Table 2**). C4BPA is regulator of Complement activation and provides an alternative pathway for activating B cells and T cells through the CD40 receptor. SERPIN B1 and IL-12 are critical factors involved in the regulation of the immune responses.

### Gene Signature Validation

**Figure 7:**
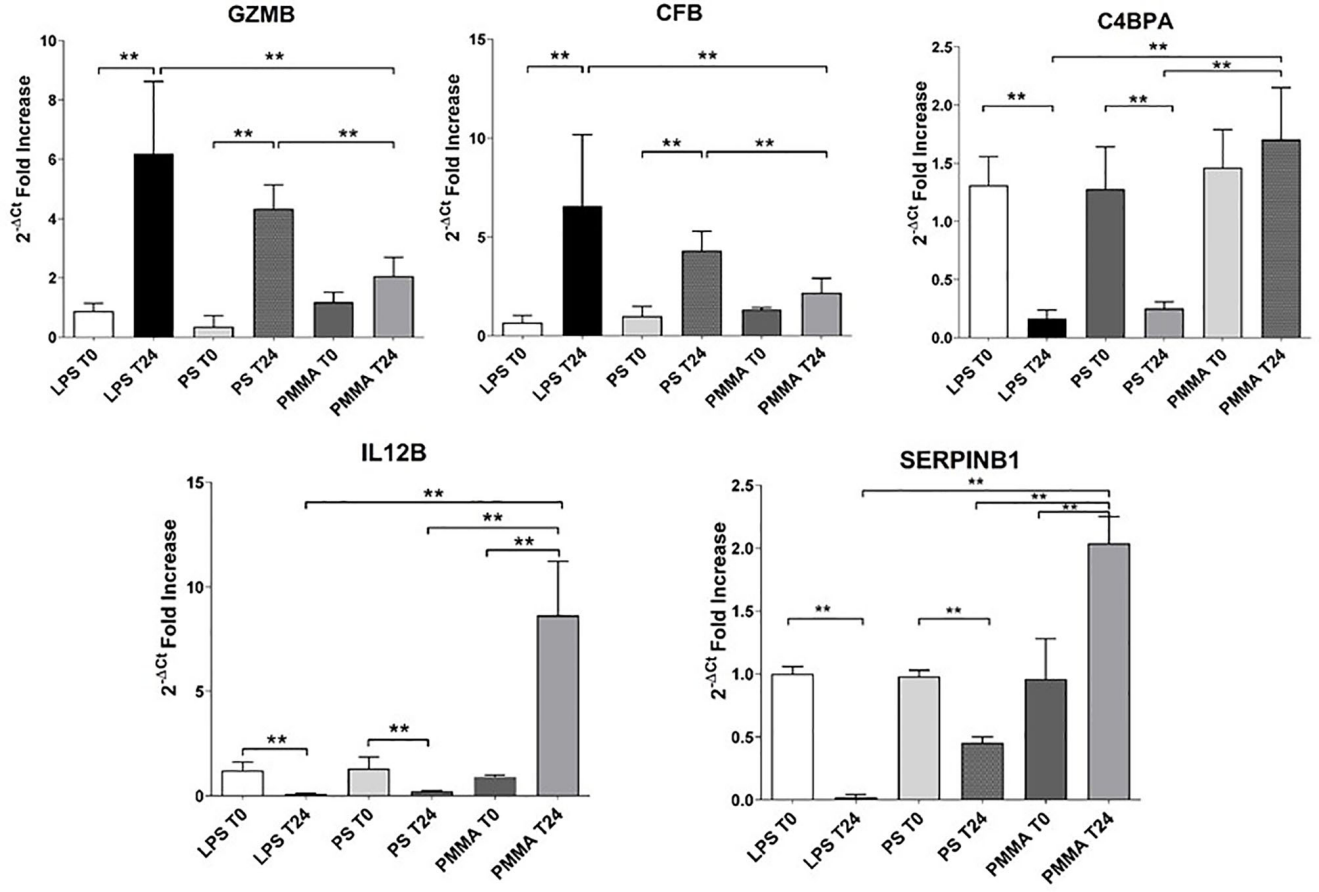
Validation of targeted genes. Real-time PCR of six genes (GZMB, CFB, C4BPA, and SERPINB1) differentially expressed in PBMC of LPS T0, LPS T24, PMMA T0, PMMA T24, PS T0, and PS T24 groups. Data are expressed as mean ± SD; **p < 0.005.

In order to validate the microarray experiments, we performed qRT-PCR analysis of the five genes in all pig groups (**Figure 7**). The qRT-PCR findings were broadly consistent with our microarray data (**Figure 6**). We observed that GZMB expression was significantly increased in LPS T24 group compared to LPS T0. This alteration trend in gene expression was also observed for the transcription of CFB gene. Next, we validated these genes also in PMMA group at T0 and T24. Interestingly, PMMA-CVVH treatment reversed LPS effects, significantly decreasing GZMB and CFB expression. On the contrary these genes were not downregulated by PS-CVVH treatment.

Then, we analyzed the effects of PMMA on C4BPA, SERPIN B1, and IL-12 gene expression. The expression of these genes was significantly reduced in endotoxemic animals respect to basal condition (LPS T0). In line with microarray results, PMMA-CVVH treatment (PMMA T24) significantly increased the expression of these genes, compared with LPS T24 group. Finally, the expression levels of these genes did not increase after PS-CVVH treatment and no significant difference was found between PS-CVVH and LPS group.

## Discussion

In this paper, we demonstrated for the first time the efficacy of PMMA-based CVVH treatment in reducing Complement activation and renal injury in a swine model of sepsis-induced AKI. Our results shed more light on the mechanism by which PMMA-based CVVH might modulate cytokine storm by preserving immunological homeostasis.

Sepsis-induced AKI is a growing health care problem and it is characterized by an overwhelmed immune response against a primary insult that become responsible for organ dysfunction and poor outcome (40). The inflammatory mediators including pathogen- and damage-associated molecular patterns bind the pattern recognition receptors (PRR), such as Toll-like receptors (TLR), expressed on the surface of immune cells, promoting a downstream cascade of signals that will amplify the immune response (1, 40, 41). Moreover, renal resident cells also expressed TLR, in particular TLR2 and TLR4, and actively participate in sepsis-induced AKI (40). During Gram-negative sepsis or endotoxin based sepsis, LPS is considered the main PAMP and is specifically recognized by TLR-4 (15) that induces the activation of pro-inflammatory and pro-fibrotic pathways in renal resident cells, contributing to fibroblasts accumulation and local inflammatory process (17, 25, 42–45). Accordingly, our data showed that the activation of TLR-4 signaling enhanced by LPS infusion, induced collagen deposition, and increased the infiltration of inflammatory cells in renal parenchyma.

The pathophysiology of sepsis is complex and involves many systems, such Complement cascade triggered both by the pathogen itself and by damaged tissue (1, 46). PTX3 plays essential role in innate immunity and Complement activation (47) and its serum level correlates with the severity of sepsis disease (33). Moreover, the end product of Complement activation, the C5b-9 complex, is associated to the development of multiple organ failure (46). Our data are in line with these findings and support the involvement of local PTX3 and Complement system in this setting.

Emerging evidences suggest that Complement activation is critical in the pathogenesis of the novel Coronavirus, SARS-CoV-2 (48). Both in bacterial and viral infections, complement system is a key player of protective immunity against pathogens, together with other innate immune system components. However, the exacerbated Complement activation induced by the pathogen, or secondarily *via* damaged host tissues become detrimental and could contribute to the maladaptive inflammatory response observed in patients with severe COVID-19 disease (9, 48). Indeed, COVID-19 patients met the diagnostic criteria for sepsis and septic shock according to the Sepsis-3 International Consensus, and the terms “viral sepsis “are more accurate to describe this complex disease (8). Therefore, it is highly plausible that the efficacy of immunomodulatory therapies in sepsis disease should be assessed in COVID-19 field.

Despite the advances in the understanding the pathogenesis of sepsis-induced AKI, the complexity of this disease might explain the failure of therapeutic intervention targeting just one of the many systems involved (49). The Surviving Sepsis Campaign Guidelines (SSCG) (50), international guidelines for diagnosis and treatment of severe sepsis/septic shock, include little description on the treatment of AKI. About renal replacement therapy (RRT), the SSCG contains weak recommendations for the choice of intermittent hemodialysis and continuous renal replacement therapy (CRRT) (4). Therefore, the hypothesis that blood purification could improve the outcome of septic AKI has attracted much attention (51). The use of PMMA membrane hemofilter in CHDF modality showed excellent removal capacity of cytokines and then it has been widely accepted in the field of chronic maintenance dialysis (52, 53). Moreover, clinical studies showed that the use of PMMA membrane hemofilter in CHDF modality reduced systemic inflammation, improved blood pressure and urine output in critically ill patients (54).

In our study, PMMA treatment induced significant modulation of hypotension, with preservation of renal function and urine output in endotoxemic animals. These data were associated with a decrease of glomerular and tubular pathological changes, inflammatory infiltrate, and early fibrosis. In addition, we observed that PMMA treatment was critical in reducing systemic Complement activation, and the acute phase protein, LBP, that it is crucial in enhancing and amplifying cellular response to endotoxin (5, 6, 18, 43).

Since Complement plays a central role in leucocyte activation (3) we investigated the modulation of Complement system in swine PBMC. Our analyses demonstrated the expression of GZMB and CFB in endotoxemic animals. These results are in agreement with those of several authors who reported increased level of GZMB and sustained Complement activation in septic patients with higher mortality and organ dysfunction (3, 55–57). When we analyzed the differentially expressed genes in PBMC of PMMA-CVVH pigs, we found that PMMA-CVVH treatment down-regulated both GZMB and CFB expression. On the contrary, these genes were not modulated by PS-CVVH treatment.

Interestingly, we also identify C4BPA, a circulating inhibitor of the classical and the lectin pathway. C4BPA provides an alternative mechanism for activating B and T cells through the CD40 receptor in a manner similar to CD40L, establishing a novel interface between B and T cell activation (58). In our animal model, we found that LPS completely downregulated C4BPA, thereby amplifying Complement system activation (59); otherwise, PMMA-CVVH was able to preserve C4BPA expression in circulating leucocytes. In addition, we observed that PMMA treatment modulate B and T cell response through the removal of the soluble CD40 (sCD40). It has been shown experimentally that sCD40 reduces immunoglobulin production and T cell activation as an immunosuppressive factor of lymphocytes activation (60). Accordingly to our results, PMMA-based filters for intermittent hemodialysis have been shown to effectively reduce the levels of sCD40 (36).

Moreover, we also observed increased serum levels of sCD40L in endotoxemic pigs and a significant reduction after PMMA treatment. As well known, sCD40L is pivotal marker of inflammation and thrombosis in several inflammatory diseases including sepsis (61, 62) and in in renal pathology (63). Therefore, PMMA was able to modulate the sCD40L/sCD40 axis, removing those mediators that are present in large number during cytokine storm and restoring homeostatic balance to assure better immune competence in septic patients. We also found increased expression of IL-12 in PBMCs of PMMA-CVVH pigs. This result is similar to the data reported by Stanilova et al. (64), who showed significant differences in the amounts of IL-12 synthesis in LPS-stimulated PBMCs between survivors and non-survivors with severe sepsis. The principal immunological function of IL-12 is to control infection increasing cellular immunity and phagocytic functions. Indeed, IL-12 induces native T-lymphocyte differentiation to type 1 T helper (Th1) cells. Th1 cells synthetize interferon gamma, which regulates macrophage and NK cell activation, induces immunoglobulin secretion by B cells, and promotes Th1 cell differentiation. Thus, increased IL-12 production in patients with severe sepsis could prevent the development of immune paralysis reducing the risk of lethal outcome (64). Accordingly, Weighardt et al. reported that impaired IL-12 synthesis augments the risk of infection after surgery and should be considered a predictive factor for the lethal outcome of postoperative sepsis (65, 66). In a prospective observational study, Chuang et al. analyzed the different mechanisms of immune dysfunction in survivors and no survivor septic patients. They observed that augmented levels of IL-12 are associated with improved general conditions in most of recruited patients and favorable outcome (67).

Another interesting gene that was upregulated in PMMA-CVVH group was SERPINB1. A recent study revealed that SERPINB1 deficiency enhances the inflammatory response to LPS, thereby exacerbating mortality and morbidity (68). Therefore, the modulation of SERPINB1 expression represents an important mechanism to minimize host damage from exacerbated inflammatory response against infection.

Finally, the gene expression analysis by qRT-PCR in PBMCs of PS-CVVH did not show any difference respect to endotoxemic animals. In conclusion, our results demonstrated that PMMA-CVVH treatment prevented the development of tubulointerstitial fibrosis, tubular damage, and inflammatory process by interfering with the activation of renal resident cells and circulating leucocytes. Indeed, PMMA showed excellent removal capacity of soluble components involved in inflammation and in immune system and reduced local and systemic Complement activation, recovering the balance between the pro- and anti-inflammatory mediators. Finally, PMMA treatment modulated gene expression in circulating leucocytes, thereby controlling complement system and innate immunity and preserving B and T cells response against infection, limiting immunological dysfunction and renal damage.

Despite the lack of a comparison of gene expression pattern between the two treatments, our results highlighted for the first time the efficacy of a new PMMA membrane in CVVH modality as a possible future therapeutic strategy with a significant impact on short- and long-term outcomes for patients with systemic inflammatory syndrome.

## Data Availability Statement

The datasets presented in this study can be found in online repositories. The names of the repository/repositories and accession number(s) can be found in the article/**Supplementary Material**.

## Ethics Statement

The animal study was reviewed and approved by the Ethical Committee of the Italian Ministry of Education, University and Research (MIUR) (Prot. n 823/2016-PR).

## Author Contributions

AS and GC planned the research, coordinated the study, performed all the experiments and statistical analyses, and wrote the manuscript. RF participated in the design of the study, performed most experiments with AS, analyzed the respective data, and contributed to draft the manuscript. CD participated in the coordination of the study and in the immunolabeling and confocal microscopy of renal sections. FSa performed microarray analysis and assisted in manuscript preparation. CC and AP assisted *in vitro* and *in vivo* experiments and in manuscript preparation. PP participated in the design of the study and helped to draft the manuscript. FSt, LL, and AC performed the animal model and helped to revise the manuscript. VC, DM, CR, MD, AL, and MZ supervised all the research. GP, GS, and LG participated in the coordination of the study and drafted the manuscript. All authors contributed to the article and approved the submitted version.

## Funding

This study was supported by University of Bari “Aldo Moro” and the Italian Ministry of Health (Giovani Ricercatori 2011-2012, GR-2011-02351027, granted to GC; Fondo Sociale Europeo, Azione I.2 “Attrazione e Mobilità Internazionale dei Ricercatori”- AIM-1810057-activity 2 granted to AS. GC was supported by an unrestricted TORAY (Toray Industries, Inc) research grant donation.

## Conflict of Interest

GC reports research grant donation from TORAY (Toray Industries, Inc), during the conduct of the study.

The remaining authors declare that the research was conducted in the absence of any commercial or financial relationships that could be construed as a potential conflict of interest.
