# Supplementary figures and images for: PMMA-Based Continuous Hemofiltration Modulated Complement Activation and Renal Dysfunction in LPS-Induced Acute Kidney Injury
Source: Front Immunol. 2021 Apr 1;12:605212. doi: 10.3389/fimmu.2021.605212 (PMC8047323; doi:10.3389/fimmu.2021.605212)

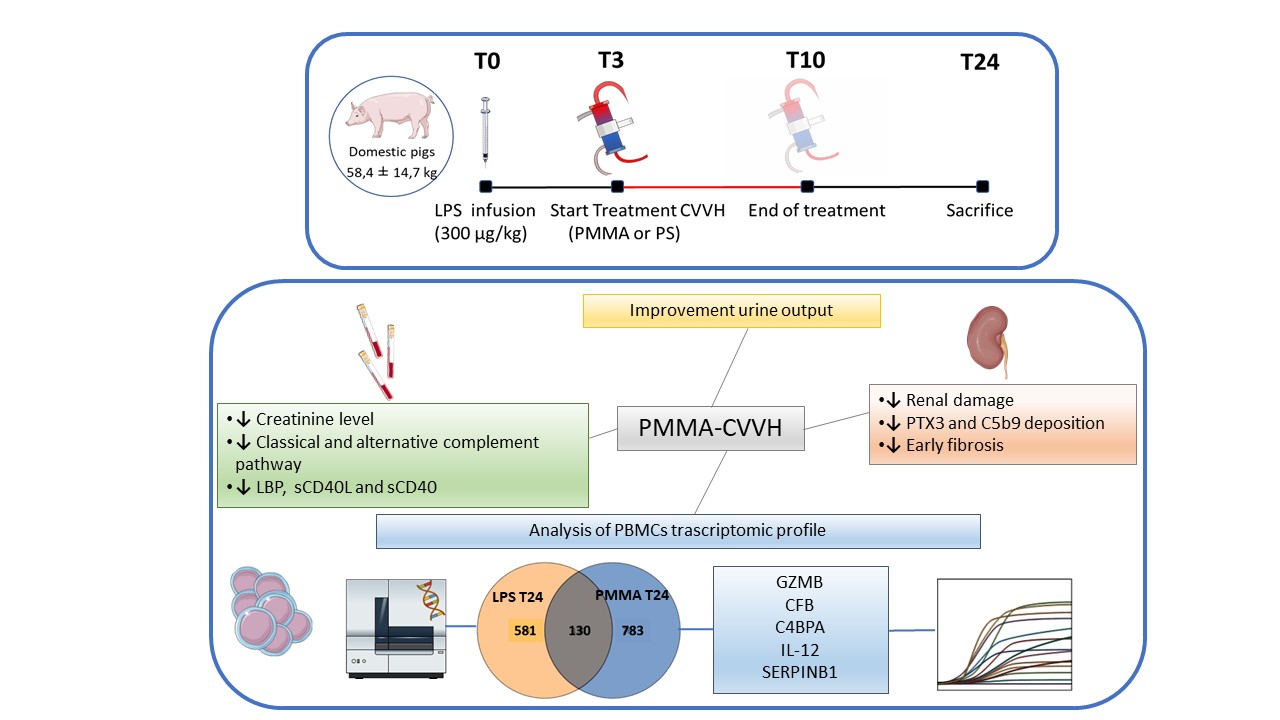

Supplement: Supplementary file 1 [file Image_1.jpeg]
